# First person – Stephan Daetwyler

**DOI:** 10.1242/bio.057851

**Published:** 2020-12-02

## Abstract

First Person is a series of interviews with the first authors of a selection of papers published in Biology Open, helping early-career researchers promote themselves alongside their papers. Stephan Daetwyler is first author on ‘Fiji plugin for annotating movies with custom arrows’, published in BiO. Stephan is a postdoctoral researcher in the lab of Reto Fiolka at UT Southwestern, Dallas, USA, investigating using light sheet and multiphoton microscopy with customized data processing and analysis to understand dynamic processes *in vivo*.


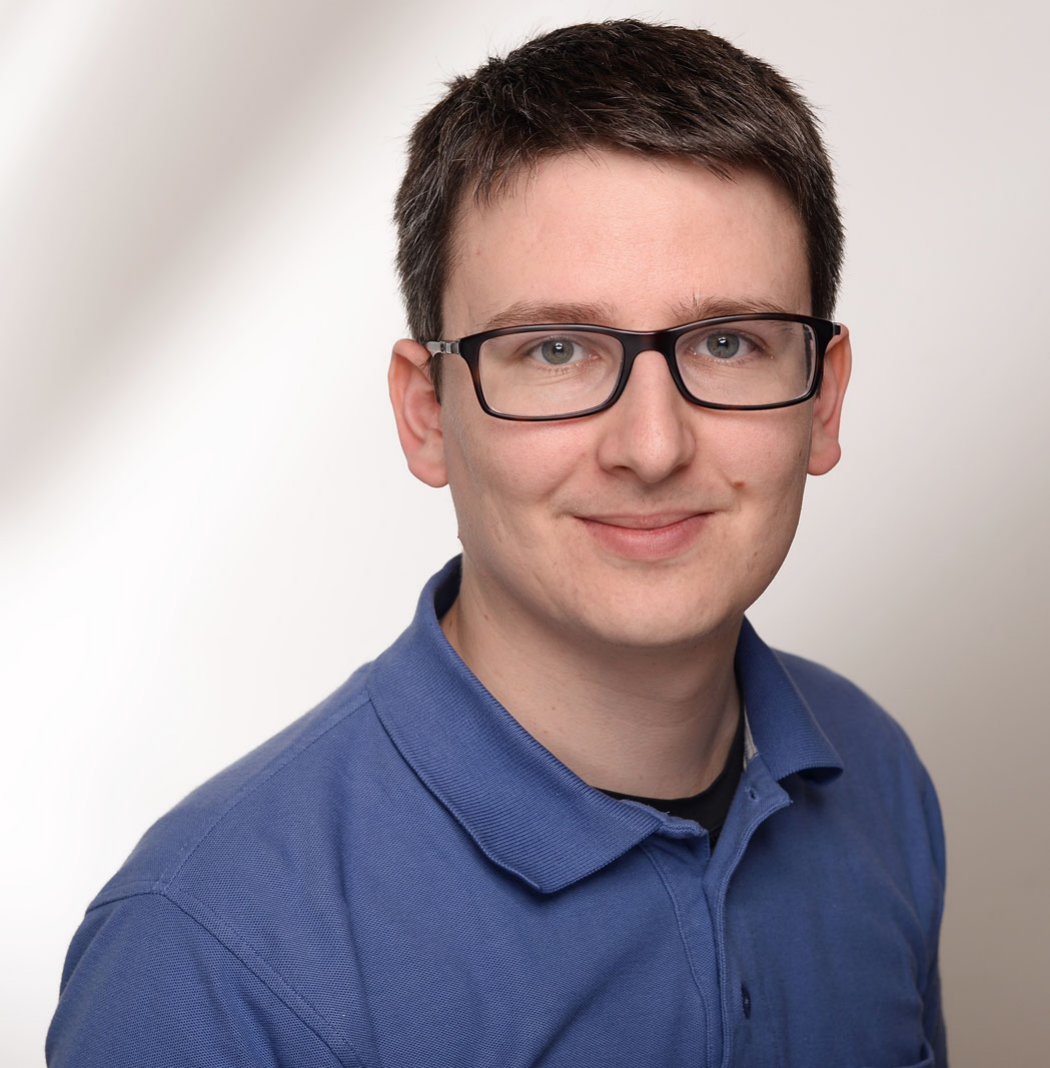


**Stephan Daetwyler**

**What is your scientific background and the general focus of your lab?**

I am currently a postdoctoral researcher in the lab of Dr Reto Fiolka at UT Southwestern and supported by an Early Mobility Fellowship of the Swiss National Science Foundation (SNSF). For my research, I am particularly interested in visualizing and understanding dynamic processes in development and disease such as cancer. The Fiolka lab and UT Southwestern provide an ideal environment to study such processes by developing and applying state-of-the art light sheet and multiphoton microscopy tools, custom data processing and analysis.

Before coming to Dallas, I received my BSc and MSc in Interdisciplinary Sciences at ETH Zurich, Switzerland, with a major in biology and physics, and joined the lab of Dr Jan Huisken at the Max Planck Institute of Molecular Cell Biology and Genetics (MPI-CBG), Germany, for my doctoral thesis. There, I implemented a dedicated multi-sample light sheet imaging, processing and analysis workflow to study vascular development, which has been published in Development (doi:10.1242/dev.173757). In collaborative work, I further applied this workflow to increase our understanding of cancer cell dissemination, initiation of metastasis, importance of blood flow on vascular remodeling and lineage tracing.

**How would you explain the main findings of your paper to non-scientific family and friends?**

Adding custom graphical symbols such as arrows, circles or arrowheads to a movie has now become much easier. Previously, researchers often tediously added graphical symbols to every single frames of a time-lapse movie. Now, our paper describes an easy-to-use and intuitive tool to add them.

**What are the potential implications of these results for your field of research?**

Annotation of movies is important for understanding them. We hope that our tool will be helpful for biologists without any coding experience to simply add custom graphical symbols such as arrows to their movies to highlight certain processes. We applied our tool already in a movie that was awarded an honorable mention in this year's 2020 Nikon small world contest (https://www.youtube.com/watch?v=LB6TJ5RmKMY).

“We applied our tool already in a movie that was awarded an honorable mention in this year's 2020 Nikon small world contest.”

**What has surprised you the most while conducting your research?**

Making a tool for general use is much harder than you think – it requires careful debugging and thinking of potential pitfalls. Therefore, I really admire the people behind large software projects, such as Fiji, who dedicate their time in providing stable and robust open source solutions to the scientific community.

**What has this influenced your research path?**

When I decided on my doctoral thesis project, I was amazed about the movies acquired on light sheet systems by my thesis mentor Dr Jan Huisken, a pioneer in light sheet microscopy. After seeing those movies, I knew that I wanted to learn how to acquire such movies, how to analyze them and how to improve the imaging techniques behind them even further.

Later, I met my current supervisor Dr Reto Fiolka at the ‘Seeing is Believing’ conference in Heidelberg. I was deeply impressed by his insights into optics ranging from high-resolution microscopy with SIM to deep tissue imaging with multiphoton microscopy, resulting in many important publications and preprints for imaging dynamic processes. These include advances such as Field Synthesis, fast remote refocusing, oblique microscopy or projection imaging.

**Figure:**
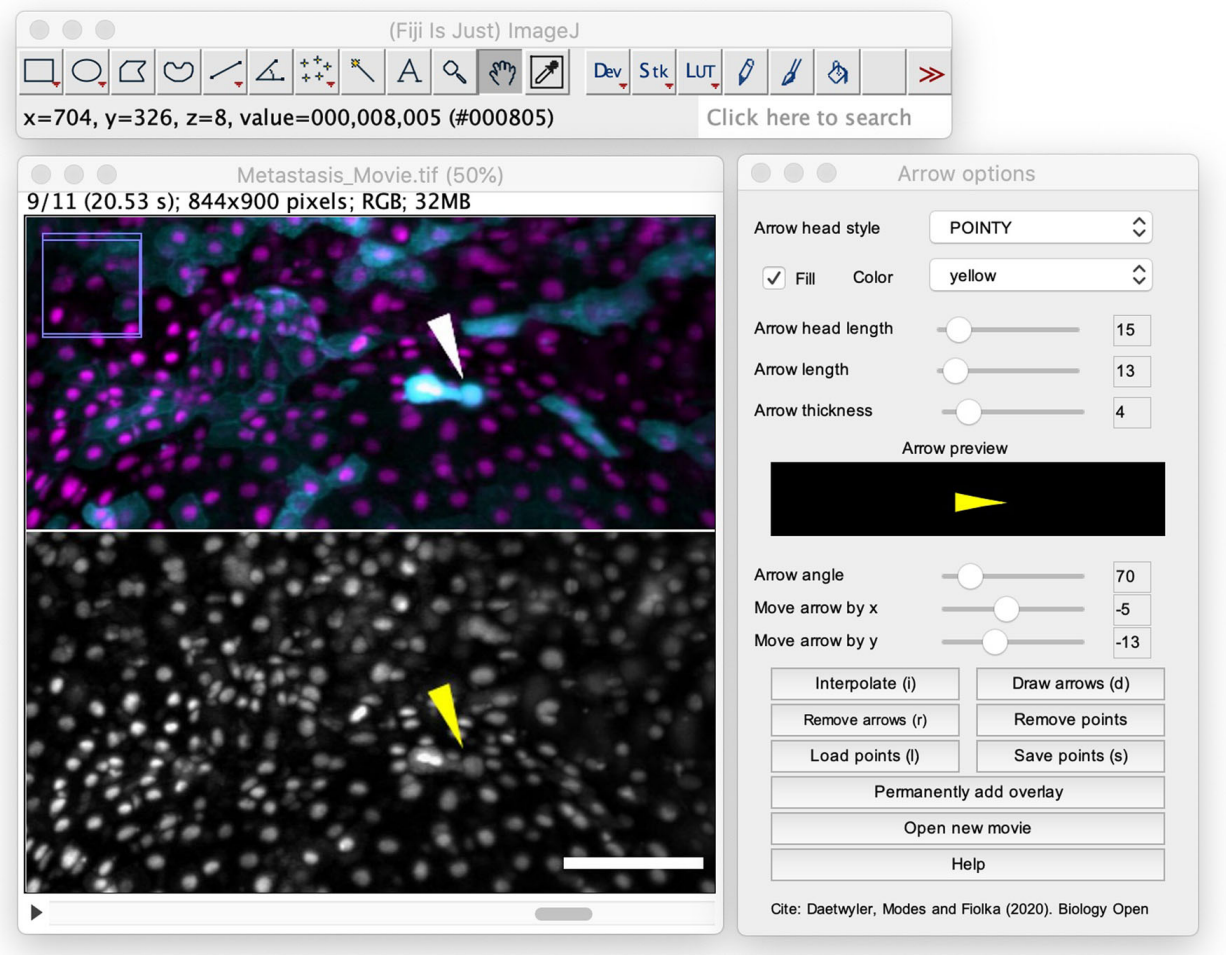
**The Fiji annotation tool in action.**

**What changes do you think could improve the professional lives of early-career scientists?**

Personally, I feel that publications and impact factor of the journals matter too much for evaluating a candidate for promotion to the next career level. While this is for sure an important aspect, other qualities such as leadership experience, experience in working with people, or outreach activities are also very important skills for a scientist managing a team of researchers as a professor. These qualities are, however, often only secondary when hiring the next assistant professor. If more engaged postdocs, experienced with leadership roles, were promoted, science overall would benefit and be more collaborative. I hypothesize that this would ultimately lead to faster progress and more discoveries.

**What's next for you?**

I am currently working on two major exciting projects to improve imaging of dynamic processes in disease and development. Hopefully, I will be able to present results of these projects next year. My long-term goal is to lead an independent research program at the interface of microscopy, computation and developmental and cancer biology.

**What are your priorities outside of your research program?**

I am very engaged in the Postdoctoral Association (PDA) at UT Southwestern as current president. This year has been a challenging year for many of us. Therefore, I have aimed at providing a nice remote work environment by quickly organizing virtual sport classes, a virtual postdoc work-in-progress seminar series, virtual coffee hours during lockdown and a virtual Postdoc Appreciation week with many activities such as a virtual mentor award and a virtual symposium. Luckily, we managed to invite this year's Nobel Prize winner, Dr Jennifer Doudna, as our keynote speaker. Moreover, I have been coordinating the postdoc response to lab occupancy rules by setting up surveys and providing inputs to the university leadership.

The health care crisis has also emphasized the need for improved working conditions of international researchers at the university. Therefore, together with the international student representatives of the graduate students and two other postdocs, I have spearheaded a ten page action plan to support our international community. We have also initiated a change of our PDA bylaws to include dedicated floating officer for Diversity, Inclusion and Equity to improve this aspect of our work.

Currently, I am organizing the first PDA inspiration award to remind us of inspiring people, and activities for the upcoming holiday season when many students might not be able to travel to their families. In addition, I am trying to emphasize the needs of our postdoc community in two 6-year plan committees.

Besides my work for the postdoctoral community, I am also active as Peer Advocate to help students in challenging life situations and in outreach as JRNLclub ambassador, a beautiful website to upload and submit movies of your research paper (https://jrnlclub.org/), and part of the community behind preLights that highlights exciting new preprints.

## References

[BIO057851C1] Daetwyler S., Modes C. D. and Fiolka R. (2020). Fiji plugin for annotating movies with custom arrows. *Biology Open* 9, bio056200 10.1242/bio.056200 33168591 PMC7725597

